# Supplementary material for: Combining the Anticancer and Immunomodulatory Effects of Astragalus and Shiitake as an Integrated Therapeutic Approach
Source: Nutrients. 2021 Jul 27;13(8):2564. doi: 10.3390/nu13082564 (PMC8401741; doi:10.3390/nu13082564)
Supplement: Supplementary file 1 [file nutrients-13-02564-s001.zip › nutrients-1266748-supplementary.pdf]

Supplementary Table S1 Anticancer properties of selected plant/mushroom bioactives *in vitro*, in preclinical animal models, and in clinical trials.

Plant/Component	Bioactives	Doses	Cell line/Animal models/Trial details	Mechanism	Comments	Reference
In vitro studies						
Astragalus membranaceous	APS (*Astragalus* Polysaccharide)	800 ug/mL<br>10 mM	MCF7, MDA-MB-231	APS inhibits Wnt/β-catenin signalling pathway (Lithium Chloride used to identify Wnt pathway)	0, 25, 50, 100, 200, 400, 800 or 1,600 μg/ml APS treated for 24 hours;<br>MTT Assay	[1]
Astragalus membranaceous	APS	50 ug/mL	A549 cells co-cultivated with mesenchymal stem cells	Reduced cell proliferation and improved cell morphology	Effects regulated by MAPK-NFkB pathway	[2]
Astragalus membranaceous	Astragaloside- IV	(10 – 160) ug/ml	Hepatocellular carcinoma cell lines – SMMC-7721 and huh - 7	Decreased expression for long non-coding RNA (Lnc RNA) and inactivated signalling of IL-11/STAT 3		[3]
Astragalus membranaceous	Astragaloside- IV	(10 – 40)umol/L	Human gastric carcinoma BGC-823 cell line	Significant increase in microRNA-214 expression and decrease in microRNA-301a expression that led to subsequent decrease in expression of SOX2 and NANOG		[4]
Astragalus membranaceous	Astragaloside- IV	0.1 mM	5-fluorouracil resistant human lung cancer cells (Bel-7402/FU)	Significant decrease in expression of multi-drug resistant gene (MDR1) by downregulating the JNK/c-Jun/AP-1 signalling pathway		[5]
Astragalus membranaceous	APS extracted at 4 and 90 ^{0}C	(25 – 800) ug/ml	MGC-803, A549 and HepG2 cells	APS4 showed higher content of (1→2,6)-α-d-Glcp compared to APS4-90 and APS90, which indicated that higher branched degree would be responsible for the stronger in vitro antitumor activity in APS4		[6]
Lentinus edodes	β-glucans fractions	1, 0.33 and 0.11 mg/mL	A549 (NSCLC), MDA-MB-231 (breast cancer cells)	Cell viability assayed – reduced by 20% at 0.11 mg/mL, 95% at 0.33 mg/mL	6 fractions from ultrafiltration process	[7]

				and 100% at 1 mg/mL (breast cancer cells)<br>Induced apoptosis at 1 mg/mL	
Lentinus edodes	Treated (with formic acid, lactic acid and boiled at 100^0C) and non-treated water soluble *lentinula edodes* polysaccharides (LEPs)	(25 – 250) ug/ml	Human breast cancer cell lines (MCF-7 and T47D)	Non-treated LEPs showed high anti-proliferative activities in MCF-7 and T47D compared to treated LEPs	[8]
Lentinus edodes	LEPs (triple helix structure) and LEPs (single flexible chain)	(5 – 500) ug/ml	Sarcoma-180 tumor cells	LEPs with triple helix structure had higher inhibition of tumor growth compared to the single flexible chain LEPs	[9]
AHCC and Wasbi combination (L edodes and Wasabia japonica)	Glucans and 6-methyl-sulfinyl-hexyl isothiocyanate	(7.5 – 500) ug/ml	MCF7, Panc02, Adenocarcinoma cell lines	Significant increase in G(0)/G(1) together with marked percentage of apoptotic cells, BAIC reduced the size and number of mamosphere formation from the stem cells	[10]
Lentinus edodes	Lacriptin – 4 domain		HepG2 cells	Inhibit growth of human HepG2 cells	[11]
Lentinus edodes	Lacriptin – 11 domain	(6.25 – 200) ug/ml	U937 (lymphoma)	Induce apoptosis	[12]
Lentinus edodes	Lacriptin – 1 domain	0 to 120 ug/mL	SGC-7901, BGC-823, SKOV-3, HepG-2, MDA-MB-231, MCF7	IC50 of 31.5 and 40.7 ug/mL for SGC-7901 and BGC-823	[13]
Lentinus edodes	LSMS-1 & LSMS-2 (polysaccharide from crude extract)	25, 50, 100, 200, 400 ug/mL	A549, SGC7901, MCF7, U937, MG63	Inhibition was maximum at 400 ug/mL	[14]
Lentinus edodes	Water extract CP	0.2, 0.5, 1.0, 2.0, 5.0 mg/mL	Hep-2 cells and MRC-5 (normal cells)	IC50 = ~1 mg/mL<br>Early apoptosis at 1 mg/mL and late apoptotic event 2 mg/mL	[15]
A. bisporus, brown A. bisporus Lentinus edodes	b-glucan	0-100 ug/mL	PC3	No significant changes in cell viability observed, dose dependent cell proliferation inhibition obtained. Ethanolic extractions reduced IL-8 production and a trend in	[16]

				VEGF secretion at higher concentration.		
Lycium barbarum	Arabinogalactan Purified fractions of *L. barabarum* crude polysaccharides (LBP-1)	50, 100, 200, 400, 800 ug/mL for 24 hours	Hepatoma cells (SMMC-7721 and HepG2), cervical cancer cells (HeLa), gastric carcinoma cells (SGC 7901), Human breast cancer cells (MCF7) and normal human liver cells (L02)	Affected cell viability in a tissue specific manner.<br>The inhibition of growth was mediated through cell cycle arrest and apoptosis.		[17]
L. edodes	LEP-1 and LEP-2	0.056, 0.112, 0.224, 0.448, 0.896, 1.792 mg/mL for 72 hours	HCT116<br>HeLa cells			[18]
A. membranaceous in combination with quercetin, dithiodipropionic acid, folic acid and curcumin (Cur)	Quercetin-3'3-dithiodipropionic acid-*Astragalus* polysaccharides-Folic acid (QDAF)@Cur	(0.5 – 20) ug/ml of Cur	MCF7 (Breast cancer cell line)	Increased intracellular uptake, tumour inhibition and apoptosis in ER-α positive cell lines		[19]
Astragalus membranaceous	Astragaloside IV	(100 – 400) uM	HCC (SK-Hep1 & Hep38 cells)	Induce extrinsic /intrinsic apoptosis, trigger G (1) arrest		[20]
Astragalus membranaceous	APS	50,100,200, 500, 1000 ug/mL and CM (Conditioned medium from macrophages)	4T1 cells	APS treated macrophages suppressed 4T1 by mitochondrial apoptotic pathway		[21]
Astragalus membranaceous	APS	5 mg/mL APS time dependent inhibition (48h)	Prostate cancer cells (PC3 and DU145 cells)	miR-138-5p/SIRT1/SREBP1 pathway in PCa	*In-vitro* and *In-vivo* Inhibited proliferation and invasion of PCa cells	[22]
Astragalus membranaceous	APS	0.25,0.5,0.75,1 and 2 mg/mL incubated for 24, 48, 72 and 96 hours	MCF7, MDA-MB-231 cells	Cell viability, migration and invasion assays demonstrates that APS interferes growth of MCF and MDA-MB-231 cells in a dose dependent manner. Mechanistically APS downregulated the expression of CCNB1 and CDC6 and		[23]

				upregulate the tumour suppressor gene p53.		
Lentinus edodes	β-glucans fractions		MDA-MB-231			[24]
Desert truffles *Terfezia boudieri*	Aqueous/methanolic extract	0.78 to 25 mg/mL incubated for 48h	T47D, MCF7, MDA-MB-231, HCT116, HeLa, Vero (normal)			[25]
Preclinical animal studies						
Astragalus membranaceous in combination with rhubarb and *Curcumae longae*	Astraagalus:rhubarb:rhihoma *Curcumae longae* (3:1:1) crude decoction in combination with small interfering RNAs (siRNAs)	6.4g/kg	Female Nude Mouse (xenograft with MCF7)	Increase the expression of Nrf2 and Suppress the expression of PI3K/AKT/mTOR signalling pathway		[26]
Astragalus membranaceous	Astragaloside - IV	160 ug/ml<br>80 ug/ml	Female C57BL/6 mice (xenograft with 3LL-luc cells)	Significant increase in expression of cytotoxic T lymphocytes while decreased expression for T regulatory cells		[27]
Astragalus membranaceous	*Astragalus* saponins (AST)	0.02,0.04, 0.08, 0.16, 0.2 mg/mL (0,12, 24,48,72h)<br><br>2% DSS (n=8, positive control), 0.1 mg/g AST (n=8, negative control) and 0.1 mg/g AST treatment combined with 2% DSS in drinking water (n=8)	Invitro (HT29, SW620) and in-vivo (DSS induced colitis mouse model – c57BL/6J mice)	IC50 for HT-29 and SW620 were 35 and 46 ug/mL<br>AST inhibits cell proliferation in a dose dependent manner and induces apoptosis (decreases Bcl2, PARP and pro-caspase-3 and increases Bax, Bak and Bad)	AST could inhibit growth and glycolysis metabolism in CRC cells *in vitro*, and attenuate the inflammatory response and tumor-like aerobic glycolysis in the DSS-induced mouse model. The findings indicated that AST may have the capacity to resist tumor-associated inflammation and maintain normal glucose homeostasis, suggesting that AST could be a novel therapeutic strategy in CRC treatment.	[28]

Mushroom mix *Agaricus blazei, Ophiocordyceps sinensis, Ganoderma lucidum, Grifola frondosa, and Lentinula edodes*	Micotherapy U-care	The blend contains 20% each of the 5 mushrooms. 16 mice received 4 mg of supplement per day – 4 healthy controls and 14 untreated	8 wk old BALB/c Syngene mice (4T1 triple negative mouse breast cancer model)	Reduced pulmonary metastasis density, decreased fibrotic response and reduced IL-6, NOS and COX2 expression		[29]
Astragalus membranaceous	APS<br>5FU<br>APS+5FU	Blank, negative (0.9% NS), APS (100 & 200 ug/Kg), 5FU (20 mg/Kg), Combined (200 mg/Kg APS + 20 mg/Kg 5FU)	Female BALB/c mice	Anticancer activity by restoring immune organs, increased cytokine release, induced phagocytosis	Along with 5FU - Immune adjuvant for chemotherapy	[21]
Astragalus membranaceous	APS	NC, LD-APS (50 mg/Kg), HD-APS (100 mg/Kg) OD – PO for 14 days	Nude mouse	Reduced tumour size and fatty acid		[22]
water extract of ginseng and *Astragalus* (WEGA)	WEGA	0,5,10,20 mg/mL – cell culture and 0, 30, 60 and 120 mg/Kg bwt for animal experimentation	A549 cells and Tumour Associated macrophages			[30]
Clinical trials						
Astragalus mongholicus and *Semen Cuscutae* or placebo			N – of – 1 with gastric cancer	Randomized double blind and controlled treatment	Symptoms improved in two patients	[31]
Antrodia cinnamomea	2.1g PS, 172 mg triterpenoids and 2.6875 g g-aminobutyric acid	2.1g PS, 172 mg triterpenoids and 2.6875 g g-aminobutyric acid	37 patients with gastric, liver, breast and crc	Double blind randomized clinical trial – 30-ay trial	No significant improvement in the combination therapy	[32]

		SD rat (toxicity)			
Ganoderma lucidum				Cochraine database – systemic review	[33]
Agaricus bisporus (white button mushroom)	6 doses from 4g/day until 14g/day		36 patients – prostate cancer	Phase-I study	[34]

Supplementary Table S2 Anticancer properties of plant and mushroom based bioactives in combination with standard chemotherapeutic agents in *in-vitro* studies, animal models, and clinical trials.

Plant/Component	Bioactive	Dose	Cell line/Animal models/Trial details	Mechanism	Comments	Reference
In vitro studies						
Astragalus membranaceous + Cisplatin	Astragaloside- IV	(10 – 40) ng/ml	Human non-small cell lung cancer cells (A549, NCI-H1299 and HCC827)	Significant increase in chemo sensitivity of cisplatin to lung cancer cells by inhibiting mRNA and B7-H3 proteins		[35]
Astragalus membranaceous Adriamycin	APS	APS (50-200 ug/mL) Adriamycin (0.1 ug/ml)	SGC-7901, SGC-7901/ADR, GES-1	APS inhibit gastric carcinoma by signalling AMPK pathway	APS 50 to 200 ug/mL plus or minus Adriamycin (0.1ug/mL)	[36]
Lentinula edodes Oxaliplatin	Lentinan Oxaliplatin	20 uM (Oxaliplatin) 800ug/ml (lentinan)	Hepatocellular carcinoma (HepG2) cells	Enhanced caspase-3 activation and reduced the expression for NF-κB and survivin		[37]
Astragalus membranaceous Taxol	AS-IV	10 – 90 uM (AS-IV) + 1-70 uM (Taxol)	MCF7, MDA-MB-231	Enhance chemosensitivity of Taxol by reversing caveolin-1 expression induced by taxol.	MTT/CFU assays	[38]
Astragalus Apatinib	APS	Apatinib 40 uM + 50, 100, 200 and 400 APS	Pancreatic cell lines, ASPC-1 and PANC-1	Inhibited cell proliferation in a dose dependent manner, increased apoptotic percentage, significantly enhanced downregulation of AKT, ERK and MMP9		[39]
Preclinical animal studies						
Astragalus membranaceous	Astragaloside IV	NS (control), Cis (3mg/Kg every 2 days PO for 14 days) or Cis+AS IV (3mg/Kg Cis + 50 mg/mL) or AS IV (50 mg/Kg IV).	BALB/c mice	Downregulated MRP2 expression in tumour cells, but not in HepG2 cells	AS IV co-administration increased chemosensitivity of Cisplatin	[40]

Lentinus edodes Oxaliplatin	Lentinan Oxaliplatin	Lentinan (25 mg/kg, i.p injection) Oxaliplatin (10mg/kg, i.p injection)	Hepatocellular carcinoma (H22) cells	Downregulated the expression of survivin, Stat3, p-Stat3 and NF-Kb (p65)	[37]
Clinical trials					
Lentinus edodes	S-1 based chemotherapy Lentinan	2 mg/Kg bwt	Chemotherapy (n=37) Chemo+Immuno (n=31)		[41]

Supplementary Table S3 Immunomodulating functions of plant and mushroom based bioactives in *in-vitro* studies, animal models, and clinical trials.

Plant/Component	Bioactive	Dose	Cell line/Animal models/Trial details	Mechanism	Comments	Reference
In vitro studies						
Astragalus membranaceous	RAP	30, 100, 300 ug/mL RAP or 1ug/mL LPS	RAW264.7 and 4T1 BMDMs (with IL-4 or LPS) BALB/c	RAP induce M1 (produced high iNOS, IL-6, TNF-a and CXL10) and skewed M2 to M1 phenotype (downregulated CD206 and upregulated CD86). Activates Notch signalling.	Reduced tumour size in mice received 4T1 grafts and Macrophages treated with RAP	[42]
Shiitake	b-glucan	100 ug/mL	PMA differentiated THP-1	Immunomodulation observed – expression of inflammation-related genes – IL-10 was highest expressed after 24 h of b-glucan.	No effect in phagocytosis index noticed except for pure Lentinan.	[43]
Astragalus membranaceous	APS in combination with cisplatin	APS- 8 and 16 mg/ml	H441, H1299, H1437 (human lung cancer cell) and LLC1 (Lewis lung cancer cell)	PBMCs from lung cancer patients (n=17)		[44]
Astragalus membranaceous	PG2 (APS)	10 ug/mL	Breast - 4T1 &colorectal - CT26. Murine WEHI-3 leukemia or murine EL4 lymphoblasts	PG2 reduced PD-L1 production which was associated with downregulation of AKT/mTOR/p7056K pathway. AKT/mTOR signal increased in murine lymphocytes when co-cultivated with PG2 treated 4T1 or CT26.		[45]
Astragalus membranaceous	APS	200 to 1000 ug/mL	RAW264.7 MCF7	Increased NO and TNF-a from macrophages and inhibited proliferation, G1 phase arrest and regulated apoptotic genes (13 fold increase in Bax/Bcl-2)	Increased NO and TNF-a expression directly blocked MCF7 cells	[6]
Preclinical animal studies						
Astragalus membranaceous	APS	50,100,200, 500, 1000 ug/mL and CM (Conditioned	4T1 cells	APS treated macrophages suppressed 4T1 by		[21]

		medium from macrophages)		mitochondrial apoptotic pathway		
Desert truffles *Terfezia boudieri*	Aqueous/methanolic extract	0.78 to 25 mg/mL incubated for 48h				
Grifola frondosa and *Lentinula edodes*	MTG404, Shiitake extract or AHCC	61.5 ug/day MaitakeGold 404, 820 ug/mL Shiitake, 820 ug Shiitake and 61.5 ug Maitake Gold 404 or 100 ug AHCC, PBS for 14 days	Female 8 wk old BALB/c mice	Phagocytic activity of peritoneal monocytes and neutrophils increased for combination, Maitake, Shiitake and AHCC in that order. Maitake and Shiitake or Maitake alone activated NK cells.		[46]
Astraalus membranaceous	Polysaccharide powder		6-8 wk old C57BL/6 mice (n=15)	PBMCs from lung cancer patients (n=17)		[44]
Clinical trials						
Lentinula edodes	Mycelia extract (LEM) – hot water extract	1.8 g/day for 4 weeks	Cancer patients	Improved quality of life, NK cell activity and immunosuppressive additive protein levels		[47]
Whole mushroom		5g (n=26) and 10 g (n=25) ODI for 4 weeks	Healthy volunteers (66 males – 21-41 years of age) Randomized parallel group study	The expression of CD69 on ɣδ-T cells increased in post treated cells than baseline cells when stimulated with a mitogen for 24 hrs.	Blood drawn before (baseline) and after 4wks of consumption – PBMC and Serum	[48]
Lentinula edodes	Lentinan b-1,3; 1.6-glucan Lentinex	2.5 mg/day	Healthy elderly individuals (42) Double blind cross over placebo controlled study	Increased circulatory B cells but no change in Ig	No change in immunoglobulins, complement proteins or cytokines	[49]
Lentinula edodes and Anthracycline based chemotherapy	*Lentinula edodes* mycelial crude extract (LEM) in combination with chemo drugs (5-FU/ cyclophosphamide/ doxorubicin/pirarubicin/ epirubicin)	LEM - 1800 mg/day for 3 wks for a total of 6 wks	Randomized double blind study (47 breast cancer patient)	QOL better for LEM group than placebo group Treg reduced for LEM group and increased for placebo group		[50]
Astraalus membranaceous	Polysaccharide powder			PBMCs from lung cancer patients (n=17)		[44]
Lentinula edodes	Mycelial extract (LEM)	1800 mg/day for 4 wks	10 patients who received DC vaccine therapy or CAT therapy	Improved QoL and increased IFN-g secretion from PBMCs (Increased FOX3P/CD4+		[51]

	expression and TGF-b excretion) (n=10)

References

1. Yang, S.; Sun, S.; Xu, W.; Yu, B.; Wang, G.; Wang, H. *Astragalus* polysaccharide inhibits breast cancer cell migration and invasion by regulating epithelial-mesenchymal transition via the Wnt/β-catenin signaling pathway. *Mol Med Rep* **2020**, *21*, 1819-1832, doi:10.3892/mmr.2020.10983.
2. Zhang, Y.M.; Liu, Y.Q.; Liu, D.; Zhang, L.; Qin, J.; Zhang, Z.; Su, Y.; Yan, C.; Luo, Y.L.; Li, J.; et al. The Effects of *Astragalus* Polysaccharide on Bone Marrow-Derived Mesenchymal Stem Cell Proliferation and Morphology Induced by A549 Lung Cancer Cells. *Med Sci Monit* **2019**, *25*, 4110-4121, doi:10.12659/msm.914219.
3. Li, Y.; Ye, Y.; Chen, H. Astragaloside IV inhibits cell migration and viability of hepatocellular carcinoma cells via suppressing long noncoding RNA ATB. *Biomed Pharmacother* **2018**, *99*, 134-141, doi:10.1016/j.biopha.2017.12.108.
4. Wang, Z.F.; Ma, D.G.; Zhu, Z.; Mu, Y.P.; Yang, Y.Y.; Feng, L.; Yang, H.; Liang, J.Q.; Liu, Y.Y.; Liu, L.; et al. Astragaloside IV inhibits pathological functions of gastric cancer-associated fibroblasts. *World J Gastroenterol* **2017**, *23*, 8512-8525, doi:10.3748/wjg.v23.i48.8512.
5. Wang, P.P.; Luan, J.J.; Xu, W.K.; Wang, L.; Xu, D.J.; Yang, C.Y.; Zhu, Y.H.; Wang, Y.Q. Astragaloside IV downregulates the expression of MDR1 in Bel7402/FU human hepatic cancer cells by inhibiting the JNK/cJun/AP1 signaling pathway. *Mol Med Rep* **2017**, *16*, 2761-2766, doi:10.3892/mmr.2017.6924.
6. Yu, J.; Ji, H.; Yang, Z.; Liu, A. Relationship between structural properties and antitumor activity of *Astragalus* polysaccharides extracted with different temperatures. *Int J Biol Macromol* **2019**, *124*, 469-477, doi:10.1016/j.ijbiomac.2018.11.156.
7. Sari, M.; Toepler, K.; Nickisch-Hartfiel, A.; Teusch, N.; Hambitzer, R. Cross-Flow Ultrafiltration Fractions of a Cold Aqueous Extract of the Shiitake Culinary-Medicinal Mushroom, Lentinus edodes (Agaricomycetes), Exhibit Apoptosis in Tumor Cells. *Int J Med Mushrooms* **2018**, *20*, 1107-11119, doi:10.1615/IntJMedMushrooms.2018028980.
8. Ziaja-Sołtys, M.; Radzki, W.; Nowak, J.; Topolska, J.; Jabłońska-Ryś, E.; Sławińska, A.; Skrzypczak, K.; Kuczumow, A.; Bogucka-Kocka, A. Processed Fruiting Bodies of Lentinus edodes as a Source of Biologically Active Polysaccharides. *Applied Sciences* **2020**, *10*, 470. doi:10.3390/app10020470
9. Zhang, L.; Li, X.; Xu, X.; Zeng, F. Correlation between antitumor activity, molecular weight, and conformation of lentinan. *Carbohydr. Res.* **2005**, *340*, 1515-1521, doi:https://doi.org/10.1016/j.carres.2005.02.032.
10. Corradetti, B.; Vaiasicca, S.; Mantovani, M.; Virgili, E.; Bonucci, M.; Hammarberg Ferri, I. Bioactive Immunomodulatory Compounds: A Novel Combinatorial Strategy for Integrated Medicine in Oncology? BAIC Exposure in Cancer Cells. *Integr Cancer Ther* **2019**, *18*, 1534735419866908, doi:10.1177/1534735419866908.
11. Guo, X.; Wang, X.; Liu, M.; Chen, L.; Chanda, W.; Pahiar, A.A.; Li, X.; Zhang, W.; Ning, A.; Huang, M.; et al. Expression and Antitumor Function of Latcripin-4 RCC1 and ANK Domain Protein on HepG2 from the Shiitake Medicinal Mushroom, Lentinus edodes C91-3 (Agaricomycetes), Transcriptome. *Int J Med Mushrooms* **2018**, *20*, 1163-1172, doi:10.1615/IntJMedMushrooms.2018028430.
12. Gao, Y.; Padhiar, A.A.; Wang, J.; Zhang, W.; Zhong, M.; Liu, B.; Kang, Z.; Wang, X.; Li, X.; Huang, M. Recombinant latcripin 11 of *Lentinula edodes* C91-3 suppresses the proliferation of various cancer cells. *Gene* **2018**, *642*, 212-219, doi:10.1016/j.gene.2017.10.080.

13. Batool, S.; Joseph, T.P.; Hussain, M.; Vuai, M.S.; Khinsar, K.H.; Din, S.R.U.; Padhiar, A.A.; Zhong, M.; Ning, A.; Zhang, W.; et al. LP1 from *Lentinula edodes* C(91-3) Induces Autophagy, Apoptosis and Reduces Metastasis in Human Gastric Cancer Cell Line SGC-7901. *Int J Mol Sci* **2018**, *19*, doi:10.3390/ijms19102986.
14. Zhang, Y.; Liu, W.; Xu, C.; Huang, W.; He, P. Characterization and Antiproliferative Effect of Novel Acid Polysaccharides from the Spent Substrate of Shiitake Culinary-Medicinal Mushroom Lentinus edodes (Agaricomycetes) Cultivation. *Int J Med Mushrooms* **2017**, *19*, 395-403, doi:10.1615/IntJMedMushrooms.v19.i5.20.
15. Finimundy, T.C.; Scola, G.; Scariot, F.J.; Dillon, A.J.P.; Moura, S.; Echeverrigaray, S.; Henriques, J.P.; Roesch-Ely, M. Extrinsic and Intrinsic Apoptotic Responses Induced by Shiitake Culinary-Medicinal Mushroom Lentinus edodes (Agaricomycetes) Aqueous Extract against a Larynx Carcinoma Cell Line. *Int J Med Mushrooms* **2018**, *20*, 31-46, doi:10.1615/IntJMedMushrooms.2018025400.
16. French, C.; Lc, C.; Clarke, S.L.; Lucas, E.A.; Smith, B.J.; Lightfoot, S.; Kuvibidila, S.R. The Inhibitory Properties of Ethanol Extracts of Some Culinary-Medicinal Mushrooms on the Secretion of Interleukin-8 and Vascular Endothelial Growth Factor by PC3 Cancer Cells. *Int J Med Mushrooms* **2019**, *21*, 645-656, doi:10.1615/IntJMedMushrooms.2019031168.
17. Gong, G.; Liu, Q.; Deng, Y.; Dang, T.; Dai, W.; Liu, T.; Liu, Y.; Sun, J.; Wang, L.; Liu, Y.; et al. Arabinogalactan derived from Lycium barbarum fruit inhibits cancer cell growth via cell cycle arrest and apoptosis. *Int J Biol Macromol* **2020**, *149*, 639-650, doi:10.1016/j.ijbiomac.2020.01.251.
18. Zhao, Y.M.; Wang, J.; Wu, Z.G.; Yang, J.M.; Li, W.; Shen, L.X. Extraction, purification and anti-proliferative activities of polysaccharides from Lentinus edodes. *Int J Biol Macromol* **2016**, *93*, 136-144, doi:10.1016/j.ijbiomac.2016.05.100.
19. Wang, B.; Guo, C.; Liu, Y.; Han, G.; Li, Y.; Zhang, Y.; Xu, H.; Chen, D. Novel nano-pomegranates based on *Astragalus* polysaccharides for targeting ERα-positive breast cancer and multidrug resistance. *Drug Deliv* **2020**, *27*, 607-621, doi:10.1080/10717544.2020.1754529.
20. Su, C.M.; Wang, H.C.; Hsu, F.T.; Lu, C.H.; Lai, C.K.; Chung, J.G.; Kuo, Y.C. Astragaloside IV Induces Apoptosis, G(1)-Phase Arrest and Inhibits Anti-apoptotic Signaling in Hepatocellular Carcinoma. *In Vivo* **2020**, *34*, 631-638, doi:10.21873/invivo.11817.
21. Li, W.; Hu, X.; Wang, S.; Jiao, Z.; Sun, T.; Liu, T.; Song, K. Characterization and anti-tumor bioactivity of *Astragalus* polysaccharides by immunomodulation. *Int J Biol Macromol* **2020**, *145*, 985-997, doi:10.1016/j.ijbiomac.2019.09.189.
22. Guo, S.; Ma, B.; Jiang, X.; Li, X.; Jia, Y. *Astragalus* Polysaccharides Inhibits Tumorigenesis and Lipid Metabolism Through miR-138-5p/SIRT1/SREBP1 Pathway in Prostate Cancer. *Front Pharmacol* **2020**, *11*, 598, doi:10.3389/fphar.2020.00598.
23. Liu, C.; Li, H.; Wang, K.; Zhuang, J.; Chu, F.; Gao, C.; Liu, L.; Feng, F.; Zhou, C.; Zhang, W.; et al. Identifying the Antiproliferative Effect of *Astragalus* Polysaccharides on Breast Cancer: Coupling Network Pharmacology With Targetable Screening From the Cancer Genome Atlas. *Front Oncol* **2019**, *9*, 368, doi:10.3389/fonc.2019.00368.
24. Morales, D.; Rutckeviski, R.; Villalva, M.; Abreu, H.; Soler-Rivas, C.; Santoyo, S.; Iacomini, M.; Smiderle, F.R. Isolation and comparison of α- and β-D-glucans from shiitake mushrooms (*Lentinula edodes*) with different biological activities. *Carbohydr Polym* **2020**, *229*, 115521, doi:10.1016/j.carbpol.2019.115521.

25. Al Obaydi, M.F.; Hamed, W.M.; Al Kury, L.T.; Talib, W.H. Terfezia boudieri: A Desert Truffle With Anticancer and Immunomodulatory Activities. *Front Nutr* **2020**, *7*, 38, doi:10.3389/fnut.2020.00038.
26. Zhang, X.Q.; Yao, C.; Bian, W.H.; Chen, X.; Xue, J.X.; Zhu, Z.Y.; Ying, Y.; Xu, Y.L.; Wang, C. Effects of Astragaloside IV on treatment of breast cancer cells execute possibly through regulation of Nrf2 via PI3K/AKT/mTOR signaling pathway. *Food Sci Nutr* **2019**, *7*, 3403-3413, doi:10.1002/fsn3.1154.
27. Zhang, A.; Zheng, Y.; Que, Z.; Zhang, L.; Lin, S.; Le, V.; Liu, J.; Tian, J. Astragaloside IV inhibits progression of lung cancer by mediating immune function of Tregs and CTLs by interfering with IDO. *J Cancer Res Clin Oncol* **2014**, *140*, 1883-1890, doi:10.1007/s00432-014-1744-x.
28. Guo, H.; Wan, B.; Wang, J.; Zhang, J.; Yao, W.; Shen, Z. *Astragalus* saponins inhibit cell growth, aerobic glycolysis and attenuate the inflammatory response in a DSS-induced colitis model. *Int J Mol Med* **2019**, *43*, 1041-1048, doi:10.3892/ijmm.2018.4036.
29. Roda, E.; Luca, F.; Iorio, C.D.; Ratto, D.; Siciliani, S.; Ferrari, B.; Cobelli, F.; Borsci, G.; Priori, E.C.; Chinosi, S.; et al. Novel Medicinal Mushroom Blend as a Promising Supplement in Integrative Oncology: A Multi-Tiered Study using 4T1 Triple-Negative Mouse Breast Cancer Model. *Int J Mol Sci* **2020**, *21*, doi:10.3390/ijms21103479.
30. Chen, Y.; Bi, L.; Luo, H.; Jiang, Y.; Chen, F.; Wang, Y.; Wei, G.; Chen, W. Water extract of ginseng and *Astragalus* regulates macrophage polarization and synergistically enhances DDP's anticancer effect. *J Ethnopharmacol* **2019**, *232*, 11-20, doi:10.1016/j.jep.2018.12.003.
31. Global Burden of Disease Cancer, C.; Fitzmaurice, C.; Abate, D.; Abbasi, N.; Abbastabar, H.; Abd-Allah, F.; Abdel-Rahman, O.; Abdelalim, A.; Abdoli, A.; Abdollahpour, I.; et al. Global, Regional, and National Cancer Incidence, Mortality, Years of Life Lost, Years Lived With Disability, and Disability-Adjusted Life-Years for 29 Cancer Groups, 1990 to 2017: A Systematic Analysis for the Global Burden of Disease Study. *JAMA Oncol* **2019**, doi:10.1001/jamaoncol.2019.2996.
32. Tsai, M.Y.; Hung, Y.C.; Chen, Y.H.; Chen, Y.H.; Huang, Y.C.; Kao, C.W.; Su, Y.L.; Chiu, H.H.; Rau, K.M. A preliminary randomised controlled study of short-term Antrodia cinnamomea treatment combined with chemotherapy for patients with advanced cancer. *BMC Complement Altern Med* **2016**, *16*, 322, doi:10.1186/s12906-016-1312-9.
33. Jin, X.; Ruiz Beguerie, J.; Sze, D.M.; Chan, G.C. Ganoderma lucidum (Reishi mushroom) for cancer treatment. *Cochrane Database Syst Rev* **2016**, *4*, Cd007731, doi:10.1002/14651858.CD007731.pub3.
34. Twardowski, P.; Kanaya, N.; Frankel, P.; Synold, T.; Ruel, C.; Pal, S.K.; Junqueira, M.; Prajapati, M.; Moore, T.; Tryon, P.; et al. A phase I trial of mushroom powder in patients with biochemically recurrent prostate cancer: Roles of cytokines and myeloid-derived suppressor cells for Agaricus bisporus-induced prostate-specific antigen responses. *Cancer* **2015**, *121*, 2942-2950, doi:10.1002/cncr.29421.
35. He, C.S.; Liu, Y.C.; Xu, Z.P.; Dai, P.C.; Chen, X.W.; Jin, D.H. Astragaloside IV Enhances Cisplatin Chemosensitivity in Non-Small Cell Lung Cancer Cells Through Inhibition of B7-H3. *Cell Physiol Biochem* **2016**, *40*, 1221-1229, doi:10.1159/000453175.
36. Song, J.; Chen, Y.; He, D.; Tan, W.; Lv, F.; Liang, B.; Xia, T.; Li, J. *Astragalus* Polysaccharide Promotes Adriamycin-Induced Apoptosis in Gastric Cancer Cells. *Cancer Manag Res* **2020**, *12*, 2405-2414, doi:10.2147/cmar.S237146.

37. Zhang, Y.; Li, Q.; Wang, J.; Cheng, F.; Huang, X.; Cheng, Y.; Wang, K. Polysaccharide from Lentinus edodes combined with oxaliplatin possesses the synergy and attenuation effect in hepatocellular carcinoma. *Cancer Letters* **2016**, *377*, 117-125, doi:https://doi.org/10.1016/j.canlet.2016.04.037.
38. Zheng, Y.; Dai, Y.; Liu, W.; Wang, N.; Cai, Y.; Wang, S.; Zhang, F.; Liu, P.; Chen, Q.; Wang, Z. Astragaloside IV enhances taxol chemosensitivity of breast cancer via caveolin-1-targeting oxidant damage. *J Cell Physiol* **2019**, *234*, 4277-4290, doi:10.1002/jcp.27196.
39. Wu, J.; Wang, J.; Su, Q.; Ding, W.; Li, T.; Yu, J.; Cao, B. Traditional Chinese medicine *Astragalus* polysaccharide enhanced antitumor effects of the angiogenesis inhibitor apatinib in pancreatic cancer cells on proliferation, invasiveness, and apoptosis. *Onco Targets Ther* **2018**, *11*, 2685-2698, doi:10.2147/OTT.S157129.
40. Qu, X.; Gao, H.; Zhai, J.; Sun, J.; Tao, L.; Zhang, Y.; Song, Y.; Hu, T. Astragaloside IV enhances cisplatin chemosensitivity in hepatocellular carcinoma by suppressing MRP2. *Eur J Pharm Sci* **2020**, *148*, 105325, doi:10.1016/j.ejps.2020.105325.
41. Ina, K.; Furuta, R.; Kataoka, T.; Kayukawa, S.; Yoshida, T.; Miwa, T.; Yamamura, Y.; Takeuchi, Y. Lentinan prolonged survival in patients with gastric cancer receiving S-1-based chemotherapy. *World J Clin Oncol* **2011**, *2*, 339-343, doi:10.5306/wjco.v2.i10.339.
42. Wei, W.; Li, Z.P.; Bian, Z.X.; Han, Q.B. *Astragalus* Polysaccharide RAP Induces Macrophage Phenotype Polarization to M1 via the Notch Signaling Pathway. *Molecules* **2019**, *24*, doi:10.3390/molecules24102016.
43. Chanput, W.; Reitsma, M.; Kleinjans, L.; Mes, J.J.; Savelkoul, H.F.; Wichers, H.J. β-Glucans are involved in immune-modulation of THP-1 macrophages. *Mol Nutr Food Res* **2012**, *56*, 822-833, doi:10.1002/mnfr.201100715.
44. Bamodu, O.A.; Kuo, K.T.; Wang, C.H.; Huang, W.C.; Wu, A.T.H.; Tsai, J.T.; Lee, K.Y.; Yeh, C.T.; Wang, L.S. *Astragalus* polysaccharides (PG2) Enhances the M1 Polarization of Macrophages, Functional Maturation of Dendritic Cells, and T Cell-Mediated Anticancer Immune Responses in Patients with Lung Cancer. *Nutrients* **2019**, *11*, doi:10.3390/nu11102264.
45. Chang, H.L.; Kuo, Y.H.; Wu, L.H.; Chang, C.M.; Cheng, K.J.; Tyan, Y.C.; Lee, C.H. The extracts of *Astragalus* membranaceus overcome tumor immune tolerance by inhibition of tumor programmed cell death protein ligand-1 expression. *Int J Med Sci* **2020**, *17*, 939-945, doi:10.7150/ijms.42978.
46. Vetvicka, V.; Vetvickova, J. Immune-enhancing effects of Maitake (Grifola frondosa) and Shiitake (*Lentinula edodes*) extracts. *Ann Transl Med* **2014**, *2*, 14, doi:10.3978/j.issn.2305-5839.2014.01.05.
47. Yamaguchi, Y.; Miyahara, E.; Hihara, J. Efficacy and safety of orally administered *Lentinula edodes* mycelia extract for patients undergoing cancer chemotherapy: a pilot study. *Am J Chin Med* **2011**, *39*, 451-459, doi:10.1142/S0192415X11008956.
48. Dai, X.; Stanilka, J.M.; Rowe, C.A.; Esteves, E.A.; Nieves, C., Jr.; Spaiser, S.J.; Christman, M.C.; Langkamp-Henken, B.; Percival, S.S. Consuming *Lentinula edodes* (Shiitake) Mushrooms Daily Improves Human Immunity: A Randomized Dietary Intervention in Healthy Young Adults. *J Am Coll Nutr* **2015**, *34*, 478-487, doi:10.1080/07315724.2014.950391.

49. Gaullier, J.M.; Sleboda, J.; Øfjord, E.S.; Ulvestad, E.; Nurminiemi, M.; Moe, C.; Tor, A.; Gudmundsen, O. Supplementation with a soluble β-glucan exported from Shiitake medicinal mushroom, Lentinus edodes (Berk.) singer mycelium: a crossover, placebo-controlled study in healthy elderly. *Int J Med Mushrooms* **2011**, *13*, 319-326, doi:10.1615/intjmedmushr.v13.i4.10.
50. Nagashima, Y.; Yoshino, S.; Yamamoto, S.; Maeda, N.; Azumi, T.; Komoike, Y.; Okuno, K.; Iwasa, T.; Tsurutani, J.; Nakagawa, K.; et al. *Lentinula edodes* mycelia extract plus adjuvant chemotherapy for breast cancer patients: Results of a randomized study on host quality of life and immune function improvement. *Mol Clin Oncol* **2017**, *7*, 359-366, doi:10.3892/mco.2017.1346.
51. Tanigawa, K.; Itoh, Y.; Kobayashi, Y. Improvement of QOL and Immunological Function With *Lentinula Edodes* Mycelia in Patients Undergoing Cancer Immunotherapy: An Open Pilot Study. *Altern Ther Health Med* **2016**, *22*, 36-42.
